# Equipping undergraduate medical and nursing students with elderly health care assessment skills at Makerere University, College of Health Sciences, Uganda

**DOI:** 10.1186/s12877-023-04561-2

**Published:** 2023-12-14

**Authors:** Noeline Nakasujja, Faith Nawagi, Blessed Tabitha Aujo, Aidah Ajambo

**Affiliations:** 1 Department of Psychiatry, College of Health Sciences, Makerere University, Kampala, Uganda; 2 Department of International Public Health, Euclid University Global Health Institute, Washington, DC USA; 3 PMTCT program, Makerere University Johns Hopkins Research Collaboration, Kampala, Uganda

**Keywords:** Health needs, Impairment, Aging, Chronic, Illness

## Abstract

**Background:**

Elderly health care training and aging science remain the least prioritized discipline of medical education in many African countries. With scant scientific evidence on elderly health care in low-income countries, coupled with limited exposure to elderly health care training, this project aimed to equip undergraduate health professional students with elderly health care assessment skills and research through an online course and a clinical placement.

**Methods:**

Students (3rd year) underwent online elderly health care training through the Alison courses published by the Advanced Learning Academy in Ireland. The students were then subjected to an online exam with a pass mark of 80%. Students were also trained on standard elderly health care assessment tools through a one-day session. For practical skills on data collection, each student assessed 15 elderly patients at the Mulago National Referral Hospital Assessment Centre. All tools once filled in were assessed for completion. A one-day reflection session was held with students, faculty and the project leads to share findings from the various tools used to assess elderly individuals. The students shared their experiences and provided feedback on the online training as well as the hardships they may have experienced while administering the tools. A certificate of participation was provided to the students at the end of the project.

**Results:**

All the students (10) gained knowledge on elderly assessment skills, the impact of aging on various body systems, and how to manage common occurrences among elderly individuals. The average score in the post-exam was 82% (standard deviation ± 2.01). All students (10) reported having had this as their first training on the assessment of functionality among elderly individuals.

**Conclusions:**

The students gained knowledge of elderly health assessments as well as the impact of aging on various body systems. They also gained insight into how to care for the elderly holistically with an added understanding of how to manage spinal and traumatic brain injuries.

## Background

Aging remains a global demographic trend of the 21st century, given the improved health service system coupled with increased life expectancy [1]. Globally, the older person (age 60 and above) contributes 11% to the general population, and this is expected to increase to 20% by 2050 [2]. Similar studies show the absolute number of older persons to have risen from 199 million to 1 billion in 1950 and 2021, respectively, and are expected to increase to 2 billion by 2050 [3].

Sub-Saharan Africa contributes 43 million, which is 5% of the global population of elderly individuals, and this figure will increase to 8.3% by 2050 [4]. In Uganda, the number of older persons has increased from 1.1 million in 2002 to 1.3 million in 2012 and is expected to increase to 5.5 million by 2050 [5]. Furthermore, life expectancy in Uganda has risen to 63 years over the past ten years [6].

Although there is no standardized cut off to define old age, the United Nations (UN), African Union (AU), and the Ministry of Gender, Labor, and Social Development (MoGLSD) use age 60 and above to refer to older persons [7].

Older persons in Uganda constitute the poorest members of society. Approximately 64% of them survive on less than US $1 a day [8]. They lack access to regular income, and the majority do not benefit from the national social security provisions since they are available to those in formal employment, yet the majority of Ugandans are self-employed. Most live in inadequate housing with others being homeless, especially in Kampala, where a few are street beggars and sleeping on the roadsides in the night, with only one meal, which leaves them emaciated and exposed to disease [9].

Elderly individuals suffer from many diseases ranging from joint pain and chronic noncommunicable diseases (NCDs) to recurrent malaria [10]. Health care is inaccessible due to various private for-profit hospital settings, which are costly in the city as well as upcountry [11]. Many suffer from multiple forms of disability. Physical disability accounts for 56% of cases, while visual impairment accounts for 39% [11]. Impairment leaves the elderly dependent on others due to the inability to engage in income-generating and self-sustaining activities, yet they need to have specialized health care [12]. They also have high levels of independence in basic activities of daily living (89%) and instrumental activities (75%) [13].

Despite the abovementioned consequences, there is scant scientific literature documented about elderly health training among undergraduate health professional students. The few studies that have been performed on elderly health have mainly focused on HIV/AIDs, antiretroviral treatment, and caregiver roles [14].

Furthermore, the Ugandan government has put in effort to address elderly health needs by putting together a policy on older persons, which as a result leads to the piloting of the Social Assistance Grants for Empowerment (SAGE) in 14 out of 112 districts in Uganda, which were all based in rural areas [10]. Under this scheme, each elderly would receive 25,000/= approximately 8 USD to cater to their daily needs [7]. The program has gone ahead to expand to 40 districts, all of which are based in the rural areas of Uganda.

Aging and its broader implications in communities are significant challenges of this century. For health services, these challenges translate into increasing demands for health workforce expertise in geriatrics and gerontology to address the health needs of elderly individuals. With hardly any exposure to elderly health care training among health professional students, this project aimed at equipping undergraduate health professional students with elderly health care assessment skills through understanding the health needs and concerns among the elderly living in Kampala, Uganda.

## Methods

### Study aim

The study aimed to equip undergraduate health professional students with elderly health care assessment skills.

### Study design

This was a cross-sectional study.

### Study setting

The research was performed at the Makerere University College of Health Sciences (MakCHS). The university is located in the capital of Uganda, Kampala, and lies 5 km north of the city centre on Mulago Hill. Undergraduate medical training programs last between three and five years.

### Ethics

The Declaration of Helsinki’s recommendations were followed when conducting the study. The Mulago Teaching and Referral Hospital’s Institutional Review Board granted ethical approval (approval code: MREC; 987). Everyone who participated signed a documented form of informed consent. Since only their online results were accessible to all students, privacy and confidentiality were guaranteed during data collection.

### Approach to training

#### Student selection

An advert was put up at the medical school notice board for interested medical and nursing students at Makerere College of Health Sciences (MakCHS) in Uganda to apply for the elderly health-training project. Students had to submit a motivation letter indicating why they were interested in participating and a short biography. Out of the 22 applications received, we selected ten students who showed up for the orientation workshop and were already in their clinical years. This group included eight medical students and two nursing students.

#### Online training session

To understand the impact of aging and how to address elderly health conditions, all students enrolled in this project undertook a free online course entitled “Elderly care and caring for the disabled,” a 02-hour introductory course on elderly health under Alison courses, published by the Advanced Learning Academy of Ireland [15].

This course was designed to equip students with basic knowledge necessary to provide adequate care to elderly individuals. The course began by helping one understand how aging affects the body, i.e., the manner in which various organs and systems throughout the body deteriorate due to the aging process, the different medical conditions that can result from such deterioration, how to identify them and how they will affect the elderly’s lifestyle [15]. Furthermore, due to accidents or medical conditions during earlier stages in life, disabilities later in old age are bound to occur. The course explored spinal cord and traumatic brain injuries, which are the most common injuries that cause individuals to require full-time, care in old age. The course concluded by reviewing the emotional impact that such traumatic injuries can have on the client, their families, and their career.

The skills necessary to support the client and their family through the care process and, if necessary, help them prepare for death were learned [15]. To establish their level of understanding upon completion, students had to attempt the online assessment included at the end of the course module. It was needed that students score 80% as a deliverable to be awarded a certificate. This score was generated by the course publisher and with an autoscoring system upon completion. Consultation with the supervising online faculty was done for any concepts that the students did not understand through email to the online course coordinator and a student‒faculty interaction page. The course duration is 1–2 h of contact time, but students can choose to do it for a given period. In this regard, the students were given one week to have completed this course, given their busy academic schedules. This course is freely available to everyone online and thus has no need for permission or clearance to undertake it.

#### Clinical exposure

The study investigators trained the students the standardized tools for elderly health care assessment used internationally to establish elderly health care needs and concerns before their clinical exposure. Each student was given a set of instruments to assess 15 elderly patients from Mulago National Referral Hospital (MNRH) Assessment Centre over a three-week period. The tools were interviewer-administered and handed over daily to the principal investigator, who would perform quality checks for completeness, give identifier numbers, and enter the information in the study database in IBM SPSS Statistics 23.0.

The instruments that were administered included the Katz index of independence in Basic Activities of Daily Living (BADL) [16], the self-rated version of Lawton Instrumental Activities of Daily Living scale (IADL) [17], the screening version of the Hearing Handicap Inventory for the Elderly (HHIE) [18], the Mini-Mental State Examination (MMSE) [19] and the Nestle Nutritional Assessment (MNA) [20].

In addition, the study team developed a survey on the social demographics of the participants. Details about the measurement are presented in another publication [13].

#### Sharing session

A half-day student sharing session was conducted for the students to share their experiences and address any questions they had after the training course. Experience, knowledge, and skills gained by the students were also assessed through a focused group discussion. A PowerPoint presentation was made to the students about the findings from the information they collected from elderly individuals, as they practiced their clinical skills with the various tools.

## Results

Ten third-year students were enrolled in the study, comprising eight males pursuing a Bachelor of Medicine and Bachelor of Surgery degree and two females pursuing a Bachelor of Nursing degree, as shown in Table 1.

**Table 1:** Participant characteristics

Characteristics	Participants n (%)
Sex	
Male Female	8 (80) 2 (20)
Year of study	
Third	10 (100)
Course studied	
Medicine and surgery Nursing	8 (80) 2 (20)

### Outcomes from the training

All data collected as per the various tools were compiled, and the findings are published. The results discussed here will focus on the approaches of training that were used and the knowledge gained by the students.

### Knowledge and skills gained by the students during the project

On prior assessment, all 10 students (100%) did not have insight into elderly health, with a significant gap in how to assess elderly health functionality status and use elderly assessment tools. Upon completion of the project, all the students [10] reported having learned the impact of aging on the various body systems, which was knowledge they had gained for the first time. They reported being confident in performing an elderly health care assessment using the tools. The average score in the final online test was 82% (± 2.01). The majority of the students [9] felt that the approach of online and clinical training enabled them to put the theory that they had learned online to practice. All the students further grounded their skills on how to use the Mini-Mental State Exam (MMSE) to assess mental status and the MNA to determine nutrition, since they had learned how to use them in other courses in the curriculum. Knowledge of how to use IADL and BADL was a new skill gained by all [10] students. All students recommended that elderly health care training be added to their undergraduate curriculum.

## Discussion

### Knowledge gained by the students

The new skills that the students learned for the very first time were the assessment of functionality using the IADL and BADL scales. Understanding elderly functionality through BADL and IADL is an essential parameter in elderly health care assessments [13]. Other skills, such as the use of MNA and MMSE, had been learned in other courses, and the training just aided in reinforcement of the skills. The impact of aging on various body systems and approaches to provide holistic care to elderly people was knowledge gained for the first time. The 8 medical students and 2 nursing students taken up for this training are the ideal students to be trained with elderly health care assessment skills given that medical doctors and nurses play the core role in health care delivery for the elderly [21].

### Approaches to teaching

The online training in this study was adequate in delivering knowledge on elderly health care given the scores each student gained (80% and above) to be considered having passed. This confirms findings from a study that was performed by Pelayo-Alvarez et al. (2013) in Spain that also found clinical effectiveness of online training in palliative care of primary care physicians (86.6%) [22]. This is a reflection that online training is practical and a useful source in medical education even in Africa, where e-learning is still being adopted as a medical training approach. Nevertheless, the challenges of limited internet access and a lack of enough computers for students remain an impediment to using this type of training in resource-limited settings [22].

On the other hand, online training provides flexibility to the students and active learner involvement, among others [22]. Flexibility is further evidenced in this study in that, despite the busy curriculum for the students at MakCHS, all students in this study completed the online training within a week and scored highly on the completion exam. However, the completion rate could have been reinforced by the fact that one had to hand in their mark score certificate to the project heads before they were allowed to proceed to attend the training on the tools and thus go for the clinical exposure. The students who applied to take up this training did it voluntarily without any reward expectation in terms of credit to be added to their academic scores. This enabled us to have a high completion rate since the students who had interest in the training are the ones that participated in this program. The online course delivery and clinical exposure were so flexible that they did not interfere with their schedules. Students had options to do this in their free time. As we had no credits/scores, we provided certificates of participation to the students and carried out a cake cutting ceremony at the end of the course to enable the students to gain motivation. Nevertheless, elsewhere, it is true that the completion level of online training is usually low, and it is a difficult method to teach clinical skills and competence to students [23].

In this study, these two barriers were addressed by a one-day face-to-face training on the tools and clinical exposure to administering all the various types of tools to 15 elderly attending care in MNRH for 02 weeks. This enabled a hybrid format of training to ensure maximum acquisition of skills. The clinical experience has, over time, been documented to allow students to gain confidence in clinical skills and, above all, improve communication skills and approaches to provide holistic care. A study performed by Gonzalo mentions that clinical exposure through ward rounds and independent clerking of patients has led to learner skill development, team building, and the promotion of patient-centered care [24]. This helps students put theory into practice and conceptualize various skills in line with patient scores with the various tools used, thus ensuring proper diagnosis [24].

All the students in this study were able to gain access to patients to assess because MNRH is the training hospital for the MakCHS and the national referral hospital of Uganda, with high patient turnover. In addition, Uganda’s life expectancy has risen to 63 years in the past decade; therefore, finding an elderly patient is no longer an infrequent occurrence [25]. The students also had ample time to practice on the tools over 2 weeks through interviewing patients. The one-day sharing session with the faculty also helped clarify all the questions or incompetence they had on using the tools and any disparities they encountered while in the field. Much as all the students recommended that elderly assessment training be incorporated into their curriculum, it is still hard to have this in place given that there are hardly any elderly health care specialists equipped with skills to train students. The curriculum in MAkCHS is too busy with hardly any gap to accommodate another module [26]. However, efforts are being made to see how best to add elderly health assessment training to the curriculum, but this is still under discussion with university management.

### Limitations

Limited interaction of the students with the training faculty as they take on the online training. However, this was addressed by the students having an interaction page where they would address questions to the faculty handling this course online.

Interruption in internet connection was rectified by providing a longer duration of time for the students to be able to complete the course.

No academic credit was ascribed to the students, as this training was not part of their curriculum. However, a certificate was given to the students documenting their participation.

There is a potential for selection bias [27] since student participation in this training was driven by their personal interest. Nevertheless, it should be noted that we extended an open invitation and actively encouraged all students to take part.

Despite the above limitations, this is the first kind of training focusing on equipping undergraduates in the MakCHS with elderly health care assessment skills. Therefore, health professional training institutions in Africa within a similar setting and where the curriculum is very busy could adopt the training formats used in this study.

## Conclusion

Students gained basic elementary skills in elderly health care assessment, with new exposure to functionality assessment of elderly individuals. There is a need to see how this training can be expanded to include more students and added to the curriculum, given that there is hardly any training in elderly health assessment, yet the life expectancy and the population of the elderly are increasing in Uganda.

## Data Availability

The datasets used and/or analysed during the current study are available from the corresponding author upon reasonable request.

## Abbreviations

BADL: Basic Activities of Daily Living
HHIE: Hearing Handicap Inventory for the Elderly
IADL: Instrumental Activities of Daily Living scale
MakCHS: Makerere University College of Health Sciences
MMSE: Mini-Mental State Examination
MNA: Mini Nutritional assessment
MNRH: Mulago National Referral Hospital
NCD: Non-Communicable Diseases
